# Fit accuracy and fracture resistance evaluation of advanced lithium disilicate crowns (in- vitro study)

**DOI:** 10.1186/s12903-024-05325-z

**Published:** 2025-01-11

**Authors:** Amro Khalil Fayed, Amir Shoukry Azer, Rewaa Gaber AboElhassan

**Affiliations:** 1 Division of Fixed Prosthodontics, Conservative Dentistry Department, Faculty of Dentistry, Alexandria University, Alexandria, Egypt; 2 Division of Fixed Prosthodontics, Conservative Dentistry Department, Faculty of Dentistry, Alexandria University, Champollion St, P. O. Box: 21527, Alexandria, Egypt

**Keywords:** Crowns, Marginal adaptation, Internal fit, Fracture resistance, CEREC tessera, IPS e.max Cad, CAD-CAM

## Abstract

**Background:**

Increasing demand for durable and aesthetically pleasing dental restorations, including laminates, inlays, onlays, and crowns, has led to advancements in all-ceramic systems, particularly with the development of advanced lithium disilicate materials. However, limited data on the fit accuracy and fracture resistance of these materials restricts their wider application in clinical restorative practices.

**Aim of the study:**

This in vitro study aims to compare the marginal and internal fit, assess the fracture resistance, and evaluate the failure modes of crowns fabricated from advanced and conventional lithium disilicate materials.

**Materials and methods:**

Thirty two (*n* = 32) crowns were fabricated and categorized into two groups based on the material used: Group (CT), where crowns were milled from CEREC Tessera (*n* = 16), and Group (EM), where crowns were milled from IPS e.max CAD (*n* = 16) using a CAD/CAM system. The marginal and internal fit were assessed digitally via a triple scan protocol. All samples were subjected to a fracture resistance test with a universal testing machine, followed by an analysis of failure modes under a stereomicroscope.

**Results:**

In the evaluation of marginal, internal and total gaps, CEREC Tessera (CT) showed slightly better fit with lower gap values compared to e.max CAD (EM). However, an independent samples t-test indicated no statistically significant differences between the two groups (*p* = 0.141, *p* = 0.471). For fracture resistance (N), the CT group demonstrated higher values than the EM group; however, the independent samples t-test indicated no statistically significant difference (*p* = 0.053). Additionally, the Chi-squared test with Monte Carlo correction revealed no statistically significant differences in the modes of fracture between the two groups (*p* = 0.484).

**Conclusion:**

Considering the limitations of this study, advanced lithium disilicate crowns demonstrated better results in terms of marginal fit, internal adaptation, and fracture resistance compared to traditional lithium disilicate crowns; however, the differences were not statistically significant. Both materials exhibited comparable fracture patterns.

**Supplementary Information:**

The online version contains supplementary material available at 10.1186/s12903-024-05325-z.

## Background

The growing patient demand for aesthetically pleasing dental restorations, has driven the innovation of advanced all-ceramic systems. The goal to replicate the shape, size, translucency, and texture of natural teeth highlights the importance of these innovations. Lithium disilicate ceramics (LDCs) have become crucial in esthetic and restorative dentistry over the past two decades [1]. Their aesthetic appeal, mechanical strength, chemical stability, and ease of processing make them versatile for numerous clinical applications, leading to their popularity among dental practitioners and technicians [1, 2]. 

With the advancement of all-ceramic systems, various materials have been improved to address their previous limitations. Lithium disilicate ceramics have enhanced mechanical properties, such as strength and fracture resistance, while new-generation zirconia has made significant progress in translucency and optical characteristics [3, 4]. 

Lithium disilicate ceramics (LDCs) are applied in various dental procedures, such as individual tooth restorations and comprehensive mouth rehabilitations. They are utilized for crowns for both anterior and posterior teeth, and fixed partial dentures up to the second premolars [5]. Furthermore, they are valued for their strength and versatility, allowing for minimally invasive or non-invasive treatments. LDCs can be fabricated into ultra-thin forms under 0.5 mm thick while preserving excellent marginal integrity and desirable optical and mechanical properties [6]. 

Fully crystallized (LDCs) provide significantly greater mechanical strength than other aesthetic materials, including feldspathic porcelains and leucite-reinforced glass ceramics. The flexural strength of fully crystallized LDCs generally surpasses 360 MPa, whereas feldspathic porcelains and leucite-reinforced ceramics have strengths between 130 and 160 MPa. This makes LDCs an optimal choice for long-lasting and visually appealing dental restorations [7, 8]. 

IPS e.max lithium disilicate can be fabricated using either lost wax hot pressing techniques or computer-aided design/computer-aided manufacturing (CAD/CAM) milling methods. These procedures can be conducted in a dental laboratory or within the dentist’s office [9]. As a monolithic CAD-CAM material, IPS e.max CAD delivers outstanding aesthetics without requiring veneering porcelain [10]. 

The composition of IPS e.max CAD includes 58–80% SiO₂, 11–19% Li₂O, 0–13% K₂O, 0–8% ZrO₂, and 0–5% Al₂O₂ [11]. It shows enhanced machinability during partial crystallization, particularly in its “blue state” before firing [12]. As it undergoes crystallization, it changes from containing 40% platelet-shaped lithium metasilicate crystals within a glassy matrix to 70% fine-grained lithium disilicate crystals [13]. The material’s robust mechanical properties, including high strength and toughness, are attributed to its high crystalline content (exceeding 60 vol%) and its interlocking microstructure. However, despite their strength, LDCs can be brittle compared to metal-based crowns, especially in thin sections or under high-stress conditions, increasing the risk of fracture, particularly in patients with parafunctional habits like teeth grinding or clenching [14, 15]. 

CEREC Tessera is a novel monolothic ceramic material that features lithium disilicate crystals measuring 0.5 μm in length, embedded in a glassy matrix, along with platelet-like lithium aluminosilicate (virgilite) crystals that range from 0.2 to 0.3 μm [16]. The manufacturer claims that this ceramic exhibits exceptional biaxial flexural strength exceeding 700 MPa [17, 18]. 

Virgilite crystals optimally form at temperatures ranging from 800 °C to 850 °C. During cooling, the differences in thermal expansion between lithium disilicate crystals and other components can lead to residual stresses or microcracks formation. However, this thermal mismatch also aids in crack tip coating, thereby increasing the material’s toughness [19]. CEREC Tessera necessitates just an additional glaze firing or matrix firing at 760 °C for 4.5 to 12 min, which can further enhance the material’s strength. In contrast, IPS e.max CAD requires a specific crystallization process [20]. 

CEREC Tessera had numerous surface fissures of varying depths prior to heat treatment according to scanning electron microscope (SEM) analysis. After firing, these fissures were absent due to the fusion of the glass matrix. Nevertheless, the quantity and size of virgilite crystals remained unchanged after the additional glaze firing [21]. Machinable lithium disilicate ceramics (LDCs) are versatile due to their strength and aesthetic appeal. However, crystallization and heat treatment after milling are essential to enhance strength and eliminate fissures caused by the milling process [22, 23]. 

Advanced lithium disilicate (CEREC Tessera) offers exceptional optical properties, enhanced by the inclusion of virgilite crystals, which improve its mechanical strength, making it comparable to the next generation of translucent zirconia. This unique combination positions CEREC Tessera as a strong alternative, blending superior aesthetics with enhanced performance. This study aims to thoroughly evaluate the viability of advanced lithium disilicate in restorative dentistry, addressing gaps in the existing literature.

Marginal and internal fit are essential factors that impact the durability and clinical effectiveness of dental restorations [24]. Larger discrepancies in these fits can result in the degradation of luting cement in the oral environment, compromising the longevity of the restorations and increasing the likelihood of failure [25, 26]. 

The study aimed to compare the marginal and internal fit, as well as evaluate the fracture resistance and failure mode, of crowns fabricated from two distinct ceramic materials: advanced lithium disilicate (CEREC Tessera) and traditional lithium disilicate (e.max CAD).

The first null hypothesis posits that there is no statistically significant difference in marginal adaptation and internal fit between the tested materials. The second null hypothesis suggests that there is no significant difference in fracture resistance between Tessera and e.max crown restorations.

## Materials and methods

The study design was in-vitro, controlled study that evaluated the marginal adaptation, internal fit, fracture resistance, and failure mode of two parallel groups. Ethical approval was obtained from the Institutional Review Board of the Faculty of Dentistry at Alexandria University (IORG: 0008839, approval no. 0557 − 12/2022). The study was carried out in the laboratory of the Conservative Dentistry Department at the Faculty of Dentistry, Alexandria University, Egypt. Power analysis was performed using a statistical software program (GPower 3.1.9.4; Henrich Heine University Dus-seldorf). Sample size was estimated assuming 5% alpha error and 80% study power [26]. Jalalian et al. [27] reported mean (SD) fracture resistance = 66.10 (51.66) and 41.14 (72.46) in case of LDS and ZLS, respectively. The mean (SD) difference = 24.96 (62.93) and the 95% confidence interval= -28.32 to 78.24. The minimum required sample size was calculated to be 14 per group increased to 15 to make up for laboratory processing errors. The total sample size = number of groups × number per group = 2 × 16 = 32 specimens [28]. The materials used in the current study are listed in Table 1.

**Table 1:** Materials used

Trade name	Manufacturer and city	Composition	Lot no.
CEREC Tessera (CT)	Dentsply Sirona, York, PA, USA	Li2O5Si2: 90% Li3PO4: 5% LiAlSi2O6 (Virgilite): 5%	16,012,393
IPS e.max CAD (EM)	Ivoclar Vivadent, Schaan, Liechtenstein	SiO2: 57.0–80.0% Li2O: 11.0–19.0% K2O: 0.0–13.0% P2O5: 0.0–11.0% ZrO2: 0.0–8.0% ZnO: 0.0–8.0% Other and coloring oxides: 0.0–12.0%	X23026
Porcelain etchant Hydrofluoric acid	BISCO-Schaumburg, USA	9.5% Hydrofluoric Acid	X-80,602 N
Bis-silane coupling agent	BISCO-Schaumburg, USA	ethanol, silane coupling agent, water	B-2221P
BisCem resin cement	BISCO-Schaumburg, USA	Base: Bisphenol-A glycidyl dimethacrylate, uncured dimethacrylate monomer, glass filler. Catalyst: Phosphate acidic monomer, glass fillers	2,200,003,884

### Preparation of the ivory teeth

**Fig. 1:**
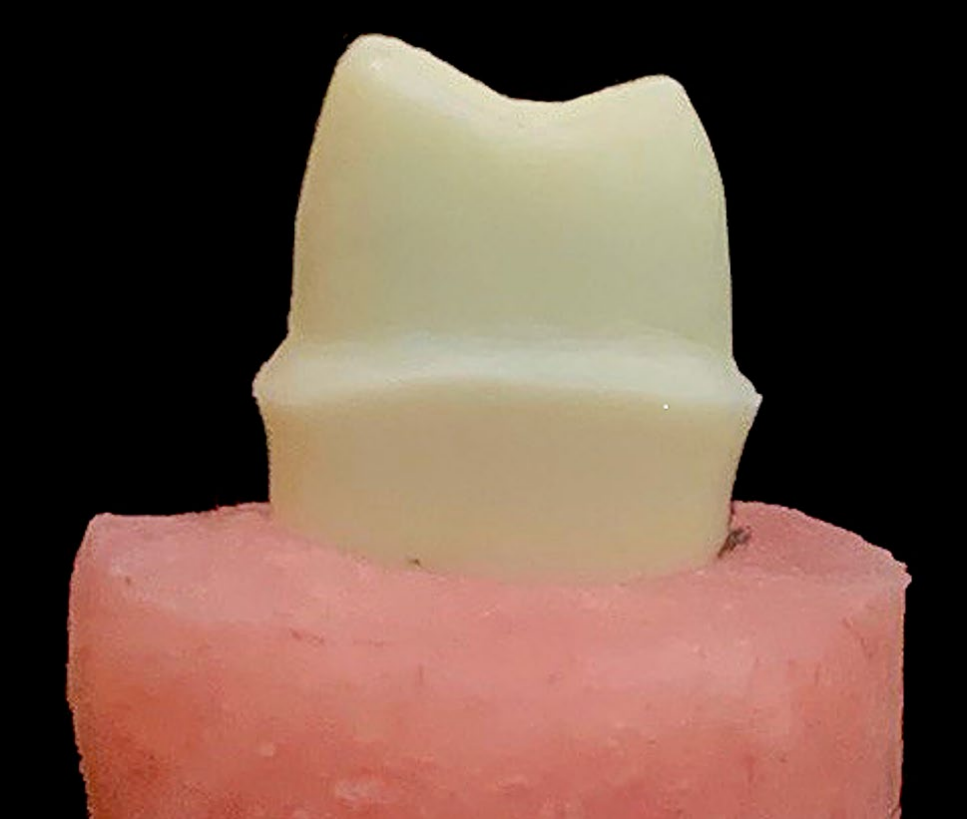
Preparation

A typodont ivory maxillary first premolar (NISSIN, Kyoto, Japan) was prepared following lithium disilicate crown guidelines, incorporating a 1.0 mm shoulder finish line, 1.5 mm occlusal reduction, and a 1.0 mm axial reduction with a consistent taper of approximately 6 to 8 degrees [29]. In order to simulate bone level, the tooth was placed with its vertical long axis in a cylindrical open mold filled with acrylic resin (Acrostone, Egypt). The mold exposed only the crown and 2 mm below the cementoenamel junction [30]. Figure 1.

### Fabrication of the epoxy resin dies

Thirty two negative replicas of the prepared master model were fabricated using duplicating addition silicone material (Dupliflex, Protechno, Spain).

Each mold was then filled with a dimensionally stable epoxy resin material (Kemapoxy 150, CMB, Egypt) with an elastic modulus similar to that of dentin, creating thirty two positive replicas of the prepared master model. These replicas were allowed to set for 24 h. A total of 32 epoxy resin dies were produced and subsequently randomly assigned into two subgroups (*n* = 16) according to the utilized ceramic materials.

### Fabrication of the restorations

**Fig. 2:**
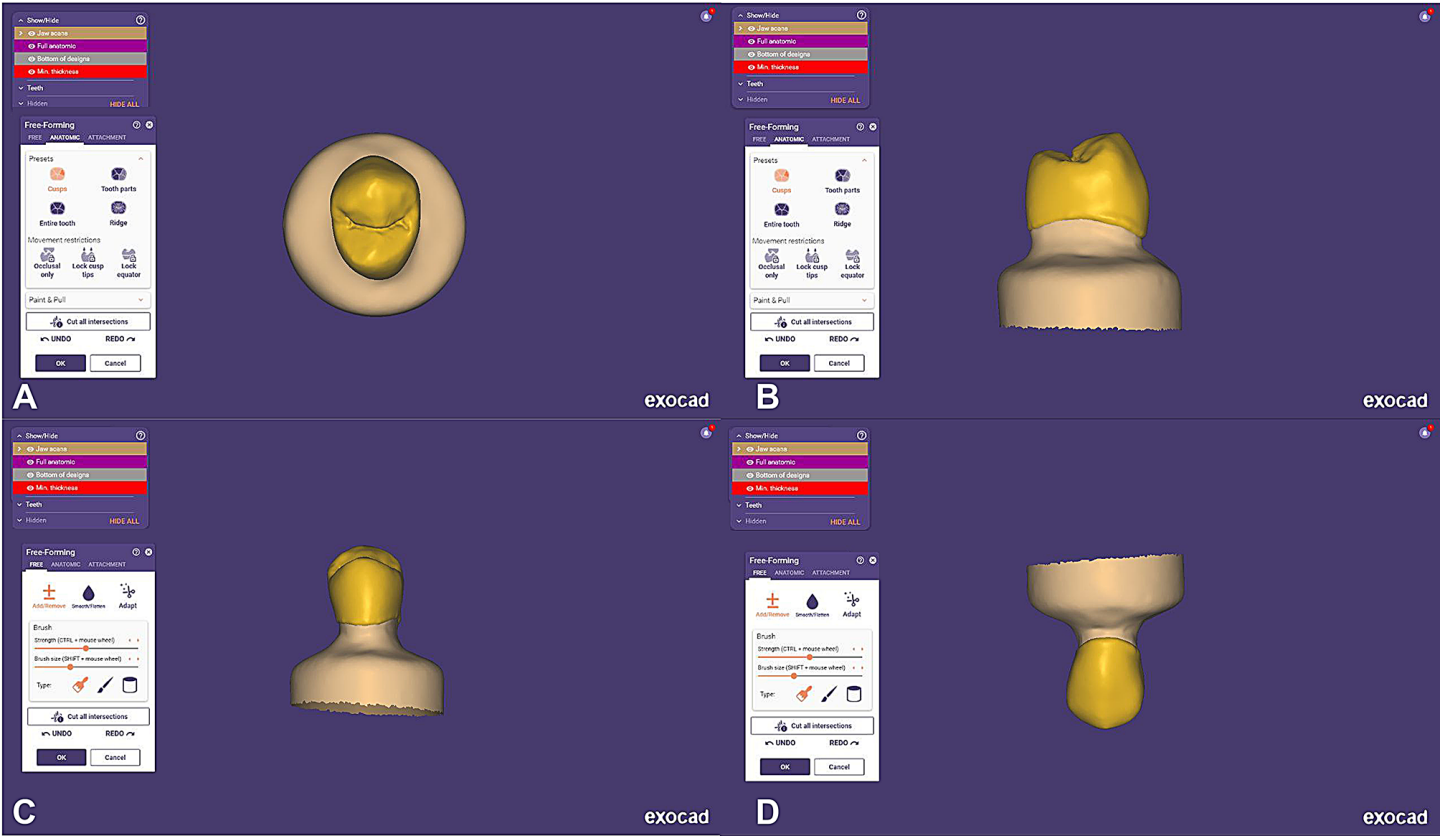
Crown design. **A**, occlusal view, **B**, proximal view. **C**, buccal view. **D**, palatal view

The epoxy resin dies were scanned using a laboratory scanner (MEDIT T710, MEDIT Corp, Seoul, Korea) in order to obtain an optical impression. Subsequently, dental CAD software (exocad Dental DB; exocad GmbH) was utilized to design full-contour anatomical monolithic crown that resembled upper first premolars. Manufacturer-recommended dimensions were followed, with the cement space set to 30 μm [31]. Figure 2. Once the design was finalized, the standard tessellation language (STL) files were transferred to a milling machine, where the crowns were fabricated using a computer-operated wet 5-axis milling device (ED5X, Emar Mills, Egypt).

Sixteen lithium disilicate crowns (EM) were milled and then glazed using FLUO Ivocolor glaze paste (Ivoclar Vivadent, Liechtenstein). The crystallization process was conducted in strict accordance with the manufacturer’s instructions, utilizing a Programat P310 furnace (Ivoclar Vivadent AG, Schaan/Liechtenstein) at a temperature of 820 °C for 20 min [32]. 

The sixteen specimens of the advanced lithium disilicate crowns (CT) underwent milling and were then subjected to an additional glaze matrix firing procedure [33]. 

### Restorations scanning

All crowns were placed on their corresponding models to verify marginal integrity, stability, and passive fit under applied force. Each crown, along with the epoxy die models, was scanned with a laboratory scanner (MEDIT T710, MEDIT Corp, Seoul, Korea). The intaglio surface of each crown and the epoxy die models were scanned with the same device [34]. This comprehensive dataset was then analyzed using specialized software (MeditDesign v2.1, MEDIT Corp, Seoul, Korea).

### Fit evaluation

**Fig. 3:**
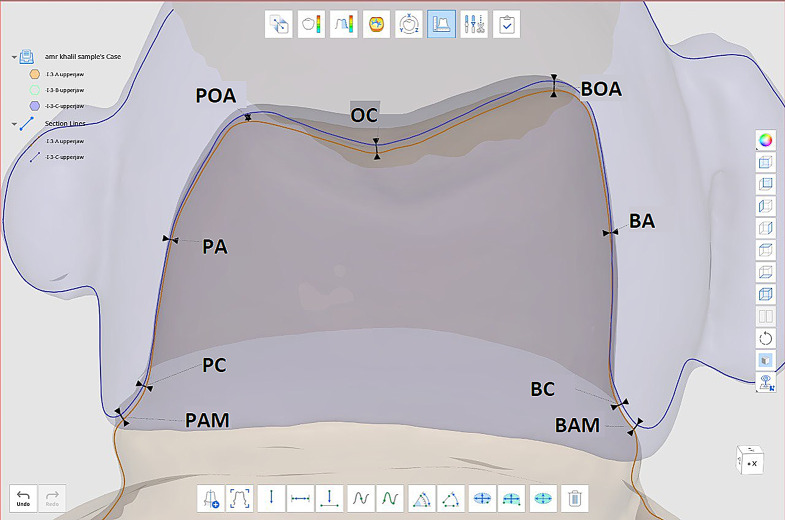
Superimposition of 3 different scans for each specimen and reference points

The scans of each specimen were superimposed to facilitate the quantitative assessment of interfacial discrepancies between the die and the restoration at predetermined landmarks, by placing composite reference points on the buccal and palatal surfaces of the crown to achieve approximate alignment, allowing for the evaluation of both marginal and internal fit [34]. Figure 3 Nine points were measured: two on the buccal surface (buccal absolute marginal point (BAM) and buccal cervical point (BC)), one on the buccal axial surface (buccal axial point (BA)), one on the buccal cusp tip (buccal occluso-axial point (BOA)), one in the center of the occlusal surface between the buccal and palatal cusps (occlusal point (OC)), one on the palatal cusp tip (palatal occluso-axial point (POA)), one on the palatal axial surface (palatal axial point (PA)), and two on the palatal surface (palatal absolute marginal point (PAM) and palatal cervical point (PC)). The two buccal points (BAM and BC) and the two palatal points (PAM and PC) represent the marginal gap, while the remaining five points indicate the internal gap. All nine points together indicate the total gap. Table 2; Fig. 3.

**Table 2:** Reference points

BAM	buccal absolute marginal point
BC	buccal cervical point
BA	buccal axial point
BOA	buccal occluso-axial point
OC	occlusal point
POA	palatal occluso-axial point
PA	palatal axial point
PAM	palatal absolute marginal point
PC	palatal cervical point

The software’s automatic alignment feature was then used to refine this alignment, utilizing advanced algorithms to accurately match the surfaces [35]. Fit analysis was performed to ensure the crown was positioned correctly on the abutment. Medit’s deviation analysis tools were employed to visualize and measure the gaps between the crown and the abutment, with the software providing a color-coded map indicating areas of tight fit, under-fit, and over-fit.

**Fig. 4:**
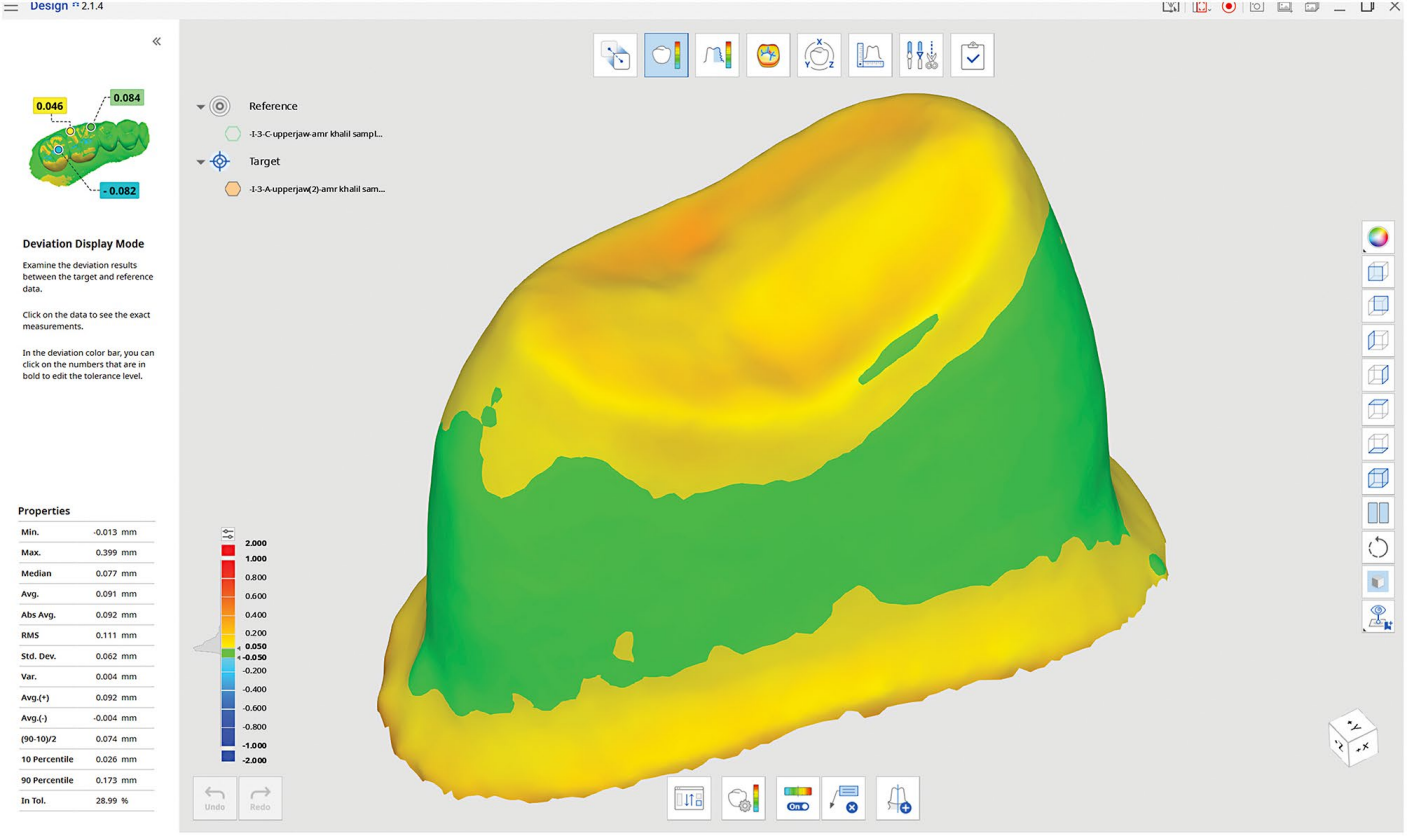
3D Color map (Heat Map)

The 3D color map (Heat map) revealed a deviation pattern illustrating the marginal and internal gaps between the tested crown and the die. In these maps, blue indicated tight contact, red signified loose contact, and yellow represented an intermediate state between red and blue, offering a qualitative assessment of the marginal fit. Figure 4.

### Crowns cementation

For both CEREC Tessera and e.max crowns, the intaglio surfaces were treated with 9.5% hydrofluoric acid (BISCO’s porcelain etchant; BISCO-Schaumburg, USA) for 20 s. They were then rigorously rinsed with flowing water and air/water spray before being dried. A single layer of Silane primer (BISCO’s porcelain primer; BISCO-Schaumburg, USA) was spread on with a fine brush for 60 s and subsequently dried with a controlled airflow. A dual-cured luting resin cement (BisCem^®^, BISCO, Inc., Schaumburg, IL, USA) was applied to the intaglio surfaces of the crowns using a mixing tip, after which the crowns were gently positioned on their respective dies with light pressure.

To ensure consistent pressure during the curing process, all specimens were subjected to a fixed force of 5 kg using a specialized static load device for 10 min, both before and during curing. A light-emitting diode (LED) curing light was then applied to the crown edges for 4 s to aid in the removal of excess cement with a scalpel blade, followed by a 40-second exposure on each surface to achieve complete polymerization.

### Fracture resistance test

**Fig. 5:**
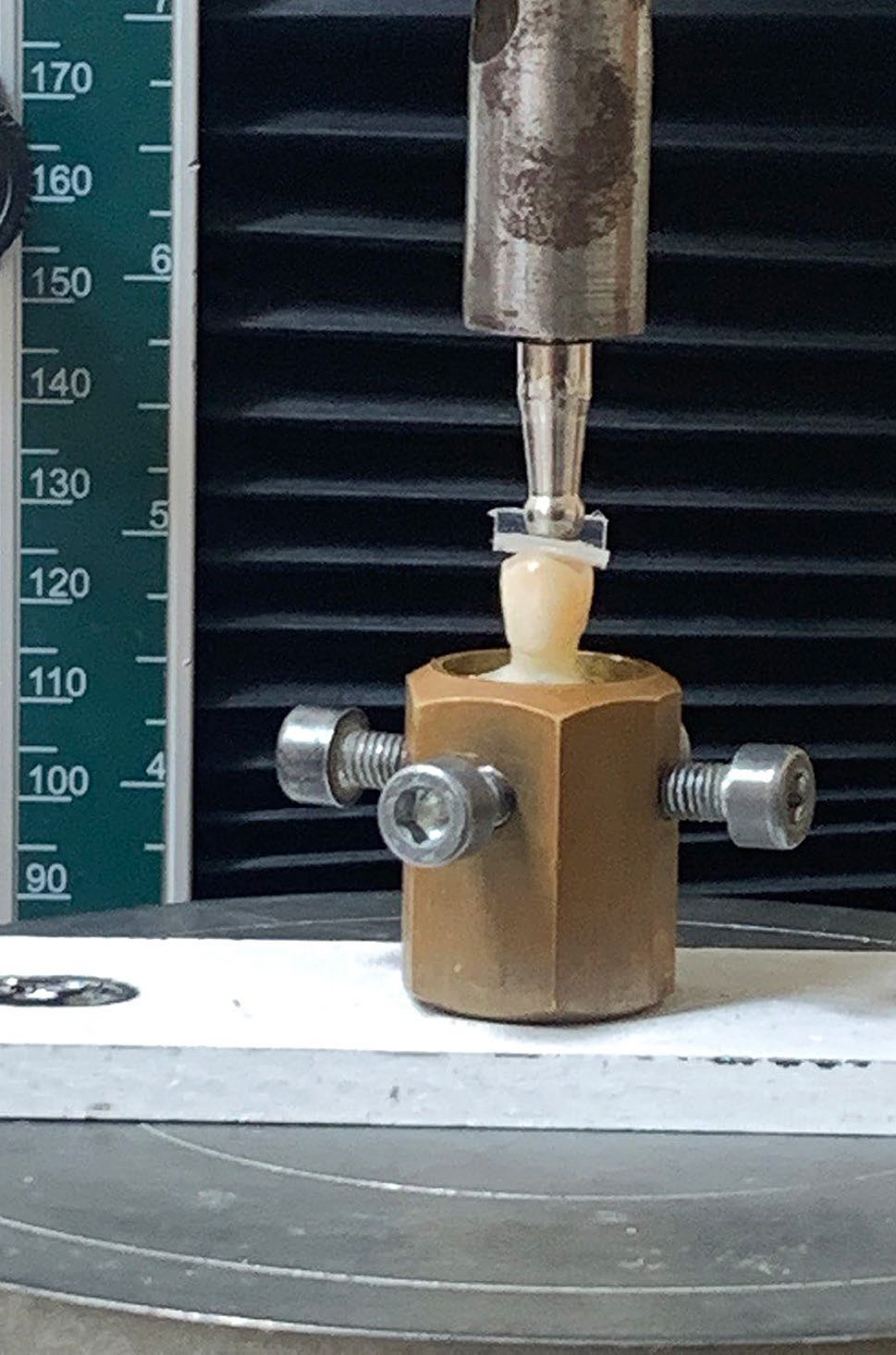
Fracture resistance test

A universal testing machine (Model 5ST, Tinius Olsen, England) was used for fracture resistance test measurements. Each specimen was affixed in a cylindrical metal holder connected to the base of the testing machine. A vertical compressive force was applied along the specimen’s vertical long axis, perpendicular to its occlusal surface, using a 4 mm stainless steel ball stylus fixed to the upper arm of the apparatus [36]. The test was performed at a cross-head speed of 0.5 mm/min. Figure 5.

To prevent unintended contact and guarantee uniform distribution of the applied force, a 0.5 mm-thick sheet of tin foil was positioned between the stylus and the specimen [37]. The load was gradually increased until fracture occurred, and the corresponding failure loads for each specimen were recorded in Newton. The data from all specimens were then compiled for statistical analysis, and the mean failure load in Newton was calculated.

### Assessment of mode of failure

After failure, the specimens underwent visual examination and were analyzed under a stereomicroscope at 20× magnification (Olympus, B061, Japan) and scanning electron microscope (SEM) at x16 and x40 magnification with an accelerating voltage of 20 KV (Jeol JSM-IT200; Jeol Ltd) to evaluate the mode of failure. The fracture modes of all specimens were evaluated and classified visually based on Burke’s classification system [38]. as:

Mode І: Minor fracture or crack in the crown.

Mode ІІ: Less than half of the crown lost. (Favorable failure)

Mode ІІІ: Midline Crown fracture. (Half of the crown lost or displaced) (Acceptable failure).

Mode ІV: More than half of the crown lost.

Mode V: Catastrophic fracture of the tooth and/or crown. (Catastrophic failure)

### Statistical analysis of the data

Data were entered into a computer and analyzed using the IBM SPSS software package, version 20.0 (Armonk, NY: IBM Corp, released in 2011). Normality was assessed using descriptive statistics, and normality tests. Qualitative data were presented as frequencies and percentages, while quantitative data were summarized using means, standard deviations, and confidence intervals. Statistical significance was determined at the 5% level.

The used tests were:

1 - **Chi-square test**.

For categorical variables, to compare between different groups.

2 - **Fisher’s Exact**.

Correction for chi-square when more than 20% of the cells have expected count less than 5.

3 - **Student t-test**.

For normally distributed quantitative variables, to compare between two studied groups.

## Results

### Marginal and internal adaptation

The values of marginal gap and internal fit points for both groups are presented in Table 3. All reference points showed that CT group had lower mean marginal gap values (*P* = 0.141), lower mean internal gap values (*P* = 0.471) and lower mean total gap values than EM group (*P* = 0.124) but with no statistically significant differences between both groups.

**Table 3:** Comparison of internal, marginal and total gaps (mm) between the two study groups

	Tessera (*n* = 16)	E-max (*n* = 16)	Difference (95% CI)	*P* value
	Mean (SD)			
BAMG	0.140 (0.020)	0.151 (0.017)	-0.026–0.002	0.091
BCG	0.107 (0.011)	0.106 (0.012)	-0.007–0.009	0.769
BAG	0.077 (0.010)	0.074 (0.011)	-0.005–0.010	0.447
BOAG	0.151 (0.022)	0.155 (0.022)	-0.020–0.012	0.604
OCG	0.127 (0.013)	0.135 (0.028)	-0.024–0.007	0.280
POAG	0.130 (0.014)	0.138 (0.023)	-0.022–0.006	0.251
PAG	0.099 (0.016)	0.093 (0.009)	-0.003–0.016	0.151
PCG	0.094 (0.013)	0.085 (0.014)	-0.001–0.018	0.094
PAMG	0.131 (0.020)	0.143 (0.026)	-0.028–0.005	0.163
Internal gap	0.117 (0.008)	0.119 (0.009)	-0.008–0.004	0.471
Marginal gap	0.118 (0.006)	0.122 (0.009)	-0.009–0.001	0.141
Total gap	0.118 (0.005)	0.121 (0.006)	-0.007–0.001	0.124

SD: Standard deviation, CI: Confidence Interval

Independent samples t-test was used

*statistically significant at p value < 0.05

### Fracture resistance test

Regarding fracture resistance, Group CT demonstrated higher fracture resistance (1249.9 ± 181.9 N) compared to Group EM (1135.1 ± 137.9 N). Although Group CT exhibited superior fracture resistance results, as shown in Table 4, the differences were not statistically significant. (*p* = 0.053).

**Table 4:** Comparison of fracture resistance between the two study groups

	Tessera (*n* = 16)	E-max (*n* = 16)
Mean (SD)	1249.9 (181.9)	1135.1 (137.9)
Mean difference (95% CI)	-1.672–231.4	
P value	0.053	

SD: Standard deviation, CI: Confidence Interval

Independent samples t-test was used

### Failure mode analysis

**Fig. 6:**
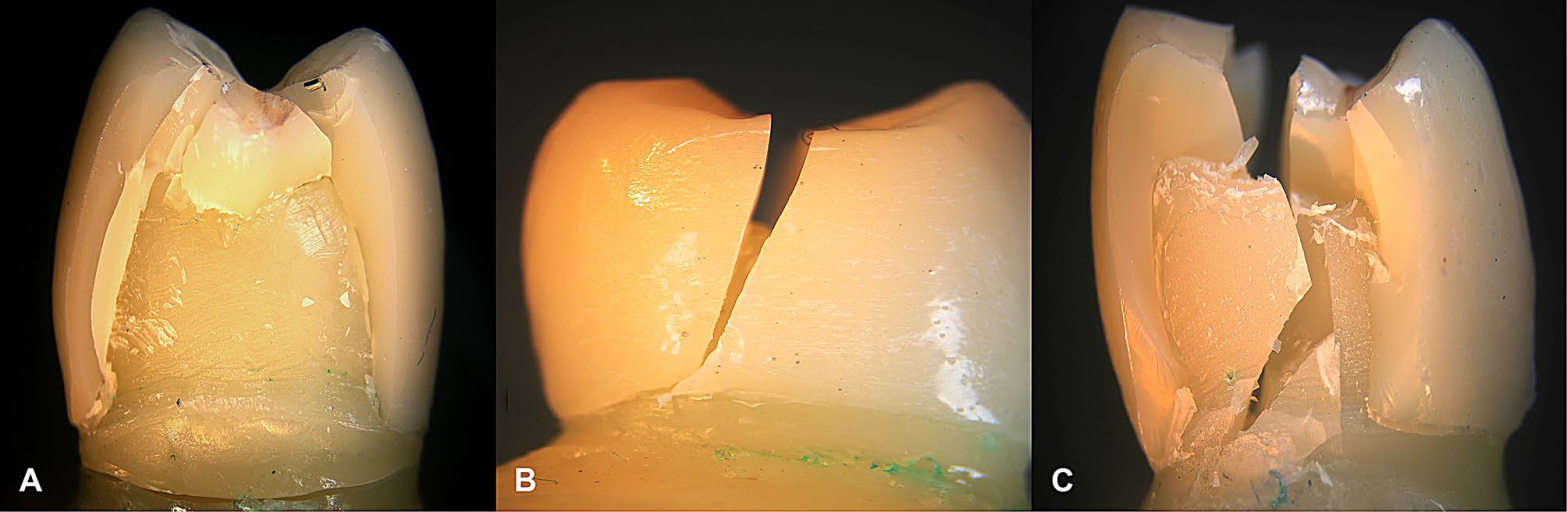
Mode of failure. **A**, Mode II; Less than half of the crown lost (Favorable failure). **B**, Mode III; Crown fracture through midline (half of the crown lost or displaced) (Acceptable failure). **C**, Mode V; Catastrophic fracture of the tooth and/or crown. (Catastrophic failure)

**Fig. 7:**
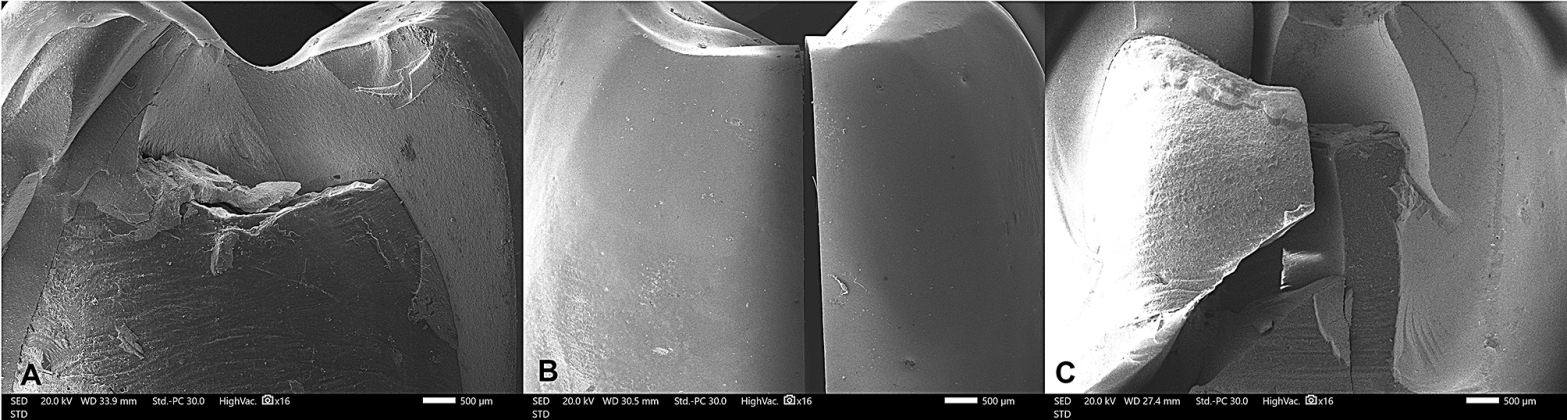
Scanning electron micrographs at 16 X magnification of three mode of failure. **A**, Mode II; Less than half of the crown lost (Favorable failure). **B**, Mode III; Crown fracture through midline (half of the crown lost or displaced) (Acceptable failure). **C**, Mode V; Catastrophic fracture of the tooth and/or crown. (Catastrophic failure)

When comparing the failure modes between the two study groups, in Group CT, fourteen specimens (87.5%) exhibited Mode II failure (favorable failure), one specimen (6.3%) showed Mode III failure (acceptable failure), and one specimen (6.3%) experienced Mode V failure (catastrophic failure). In contrast, all specimens in Group EM (100%) demonstrated Mode II failure (favorable failure). The differences between both groups were not statistically significant (*p* = 0.484) Table 5; Figs. 6 and 7.

**Table 5:** Comparison of failure mode between the two study groups

	Tessera (*n* = 16)	E-max (*n* = 16)
	*N* (%)	
Mode II (favorable failure)	14 (87.5%)	16 (100%)
Mode III (acceptable failure)	1 (6.3%)	0 (0%)
Mode V (catastrophic failure)	1 (6.3%)	0 (0%)
Chi-square test P value	χ^2^: 2.000 ^FE^p: 0.484	

χ^2^: Chi square test FET: Fisher Exact test

p: p value for comparing between Group (CT) and Group (EM)

Chi-square with Monte-Carlo corrected p value was used

## Discussion

This in-vitro study evaluated the marginal and internal fit, examined the fracture resistance, and analyzed the mode of failure of advanced lithium disilicate (CEREC Tessera) and traditional lithium disilicate (e.max CAD) crown restorations fabricated using CAD/CAM technology. In the present study, both null hypotheses were accepted, as the results showed no statistically significant differences between CEREC Tessera and e.max CAD crowns in terms of marginal adaptation, internal fit, or fracture resistance. CEREC Tessera, a newly developed CAD/CAM block, delivers exceptional aesthetics, offers up to 32% greater strength compared to other glass ceramics, and reduces processing time by up to 44% [5]. 

With the development of CAD/CAM techniques, evaluation of fit accuracy techniques, including the double-scan and triple-scan protocols, are considered as recent digital variations of the traditional replica technique [39]. The comprehensive triple-scan protocol, utilizing specialized software, offers several features. It provides precise measurements for accurate crown fit assessments, reduces human error from manual measurements, and ensures consistent, reliable results. Additionally, it saves time compared to traditional replica techniques [24, 40]. 

The fracture resistance test results from the present study revealed that the CEREC Tessera (CT) group exhibited a higher mean fracture strength compared to the e.max CAD (EM) group. The difference can be due to the distinct microstructure of the CEREC Tessera ceramic material, which likely contributes to its enhanced strength. Clinically, masticatory forces generally range between 600 N and 800 N, but these values can exceed this range in patients with bruxism, particularly in the molar region. The study’s findings demonstrated that the load required to fracture any of the crowns tested surpassed the typical clinical masticatory forces, indicating the suitability of both materials for clinical use in high-stress areas [41, 42]. 

To ensure standardization, all dies were duplicated from the same typodont tooth. The epoxy resin dies were selected for their dimensional stability and a modulus of elasticity of 11.8 GPa, comparable to that of dentin at 18 GPa. Crown restorations were bonded to these epoxy resin dies using a dual-cured universal adhesive resin cement [43]. 

In evaluating marginal and internal fit, this study found no significant differences between the CEREC Tessera (CT) and e.max CAD (EM) groups. The (CT) group exhibited a marginal mean gap value of 0.118 ± 0.006 mm and a mean internal gap value of 0.117 ± 0.008 mm. Similarly, the (EM) group showed a marginal mean gap value of 0.122 ± 0.009 mm and a mean internal gap value of 0.119 ± 0.009 mm. These results are consistent with the findings of Perez et al. [17], who observed that while CEREC Tessera demonstrated slightly lower mean marginal fit values than IPS e.max CAD, the difference was not statistically significant. Another study by Demirel et al. [44] further supports our findings, reporting mean gap values of 108.6 ± 3.8 μm for e.max CAD and 102.8 ± 4.8 μm for Tessera, with no significant differences between the groups after crystallization. Their study also noted a considerable reduction in the internal gap of e.max CAD after crystallization, whereas no such effect was observed for Tessera.

In contrast to our findings, Ferrini et al. [26] evaluated the marginal gap of lithium disilicate crowns produced via CAD-CAM on a custom chrome-cobalt (Cr-Co) implant abutment. They recorded an average marginal gap of 62.28 ± 51.8 μm for CEREC Tessera crowns, which is lower than the values observed in our study. This discrepancy may result from using a chromium-cobalt abutment with a chamfer finish line and employing scanning electron microscopy for measurement. Similarly, Kojima et al. [45], using microfocus X-ray CT, observed that e.max crowns had a significantly smaller average gap distance compared to CEREC Tessera crowns. However, after heat treatment, a significant increase in the marginal gap of e.max crowns was observed, whereas the marginal gap of CEREC Tessera crowns decreased. This difference may be attributed to the dynamic softening of e.max, which likely deformed during heat treatment as the temperature surpassed its softening point.

Other studies [25, 46, 47] comparing crowns fabricated from E.max CAD and E.max Press have used various techniques, such as triple scanning, light microscopy, and micro-CT X-ray, to evaluate marginal and internal gaps. These studies have reported different values, indicating that material choice is not the sole factor influencing the adaptation and marginal fit of CAD-CAM-milled crowns.

Fracture resistance tests are essential in evaluating dental crowns, these tests ensure that the crowns are able to withstand the forces of chewing and biting in the oral environment to provide long-term functionality for the patient [48]. The current study showed that the mean fracture resistance for (CT) group was 1249.9 ± 181.9 N, while the (EM) group had a mean fracture resistance of 1135.1 ± 137.9 N, with no significant difference between them. The lack of statistically significant difference in fracture resistance can be attributed to several factors. Material composition plays a key role, as both CEREC Tessera and e.max CAD are lithium disilicate glass ceramics. CEREC Tessera incorporates zirconia for enhanced mechanical properties, while e.max CAD offers a balance of strength and aesthetics. Both materials are designed for high fracture resistance, making them ideal for areas subject to significant masticatory forces. Additionally, both materials exhibit high flexural strength—360–400 MPa for e.max CAD and up to 700 MPa for CEREC Tessera—as well as high compressive strength, contributing to their durability. The microstructural advancements, such as zirconia reinforcement in CEREC Tessera and optimized crystal growth in e.max CAD, enhance fracture toughness and help prevent crack propagation. Furthermore, the standardized and controlled testing conditions ensured that the inherent properties of both materials showed similar performance due to their high-quality composition and design.

**Fig. 8:**
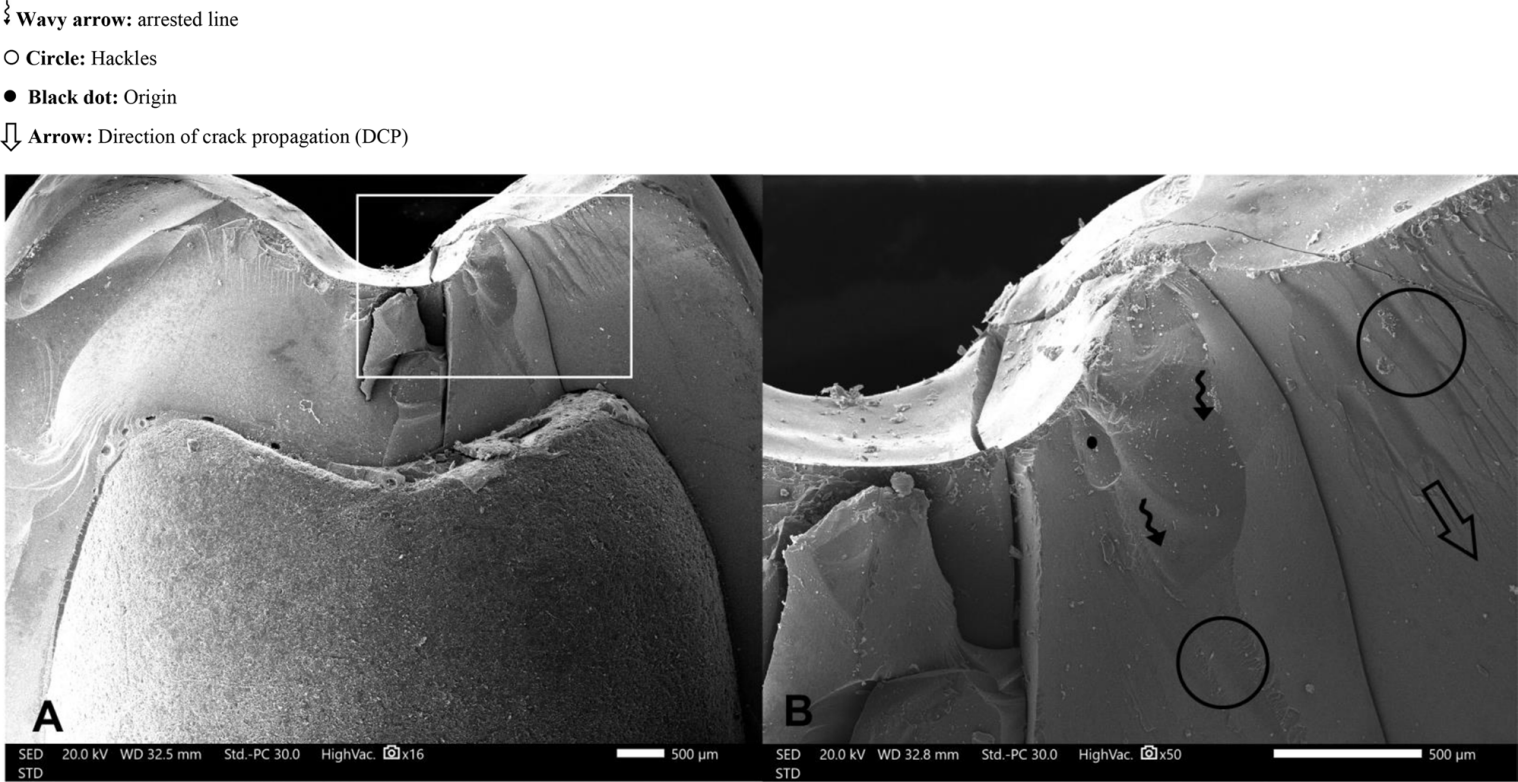
**A**) Scanning electron microscope at 16 X magnification, **B**) Scanning electron microscope at 40 X magnification. (Magnified view of the square highlighted region)

**Fig. 9:**
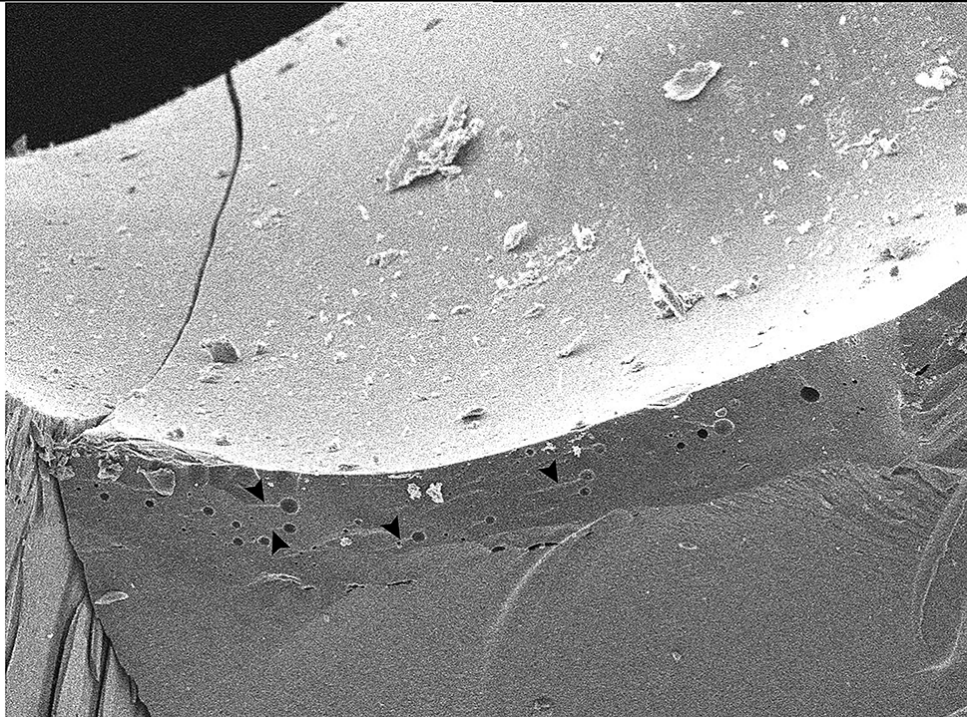
Scanning electron microscope, wake hackles appearance related to the voids present on the surface of crown. Arrow head: wake hackles

In this study, the majority of specimens exhibited Mode II (favorable) failure, with one specimen displaying Mode III (acceptable failure) and another undergoing Mode V (catastrophic failure). This distribution underscores the advanced mechanical properties of lithium disilicate, contributing to enhanced fracture resistance and predictable, clinically acceptable outcomes. The occurrence of catastrophic failure may be associated with microstructural imperfections, such as voids identified in SEM analysis Figs. 8 and 9, or potential laboratory and sintering inaccuracies during the milling process, highlighting the influence of material and processing variables on failure patterns.

Some studies propose that self-adhesive resin cementation has a little impact on the fracture resistance of glass-ceramics. However, evidence indicates that bonded all-ceramic restorations generally exhibit higher fracture resistance than those cemented conventionally. This increased resistance is likely due to the elastic properties of resin cement, which enable it to deform under stress and thereby enhance the overall fracture resistance [49–51]. 

In alignment with our findings, Balladares et al. [52] reported no significant difference between crystallized CEREC Tessera and crystallized e.max molar crowns regarding fracture resistance. The relatively low values could be due to their methodology, as they did not bond the crowns to the die, focusing on obtaining accurate and detailed data purely on the mechanical performance. Additionally, Rosentritt et al. [53] investigated the fracture resistance of crown restorations made from CEREC Tessera and e.max CAD using various cements. They found no significant difference in fracture force between both materials. When adhesive resin was used for cementation, the mean fracture resistance was 2101 ± 752 N for CEREC Tessera, which was lower than the 2529 ± 468 N for e.max CAD. The increased fracture resistance compared to the present study may be attributed to the utilization of human molar teeth in their study, providing a more accurate representation of clinical conditions and enhancing bonding for a more robust ceramic-tooth interface.

In contrast, Jurado et al. [54] reported a significant difference in fracture resistance between mandibular molar crowns made from CAD-CAM lithium disilicate with virgilite (CEREC Tessera) and those made from traditional lithium disilicate (e.max CAD). Furthermore, Nouh et al. [55] reported that advanced lithium disilicate crown restorations demonstrated a mean fracture resistance significantly higher than that of traditional lithium disilicate. However, despite these notable differences, the mean fracture resistance values align closely with those observed in our study, which may be attributed to the use of epoxy resin dies as abutments.

Jurado et al. [56] found that traditional lithium disilicate ceramics showed higher fracture resistance than those containing virgilite. The results of the study could be influenced by the use of resin dies, which may affect the fracture resistance measurements. In a separate study, M. Mohammed et al. [57] identified a statistically significant difference in fracture resistance between endocrowns made from CEREC Tessera and e.max CAD. Their findings showed that endocrowns created with CEREC Tessera exhibited a higher average fracture resistance than those fabricated from e.max CAD.

Other factors, such as preparation design and cement space, can affect the fracture resistance mean values. Hamza et al. [58] found the mean fracture resistance of lithium disilicate crowns (e.max CAD) to measure 1565.2 ± 89.7 N. These high values may be due to the epoxy resin dies, with a flat occlusal surface and an 80 μm cement space. This design likely increased the force required to fracture the tooth-restoration complex. Additionally, the 80 μm cement space provided greater strength compared to the 30 μm space used in our study [59]. 

The absence of a statistically significant difference in fracture resistance between CEREC Tessera and e.max CAD premolar crowns indicates that both materials possess high mechanical strength and durability. Their advanced compositions and microstructural designs enable them to perform similarly well under the conditions tested. This finding underscores the suitability of both materials for use in demanding clinical situations where high fracture resistance is required.

This study identified several limitations, including its in vitro design and the use of epoxy resin dies instead of natural tooth abutments. These factors may impair bonding and influence the fracture resistance results. Furthermore, most in vivo fractures are likely attributed to fatigue conditions instead of the compressive load primarily examined in this study. Further studies are needed to perform fractographic analysis of fracture patterns and to investigate the microstructure of fracture surfaces in advanced lithium disilicate. Additionally, comparative studies should be undertaken to evaluate advanced lithium disilicate against other emerging materials, such as new generations of zirconia and composite resin, in terms of their mechanical properties and optical performance.

## Conclusion

Considering the limitations of this in vitro study, the subsequent conclusions can be inferred:

1. Advanced lithium disilicate demonstrates favorable characteristics regarding mean marginal gap, total gap, and internal adaptation, exhibiting comparable performance to e.max CAD.
2. While CEREC Tessera crowns exhibited higher mean fracture resistance values than e.max CAD crowns, the difference was not statistically significant, indicating equivalent mechanical performance.
3. The mode of failure observed in both materials showed no statistically significant differences, suggesting similar behavior under mechanical stress.
4. All evaluated parameters for both materials fell within clinically acceptable ranges, supporting their reliability and suitability for clinical applications in restorative dentistry.

## Electronic Supplementary Material

Below is the link to the electronic supplementary material.

Supplementary Material 1

## Data Availability

The datasets generated and analyzed during the current study are available from the corresponding author on reasonable request.

## Abbreviations

°C: Centigrade degrees
CAD-CAM: Computer aided design and computer aided manufacturing
Cr-Co: Chrome-cobalt
CT: Cerec Tessera
EM: E-max Cad
IBM SPSS: Software for advanced statistical analysis
LDCs: Lithium Disilicate
min: Minute
mm: Millimeter
N: Newton
sec: Second
STL: Standard tessellation language

## References

[1] Fu L, Engqvist H, Xia W. Glass–ceramics in dentistry: A review. Materials. 2020;13:1049. 32110874 10.3390/ma13051049 PMC7084775

[2] Holand W, Beall GH. Glass-ceramic technology. Wiley; 2019.

[3] Kongkiatkamon S, Rokaya D, Kengtanyakich S, Peampring C. Current classification of zirconia in dentistry: An updated review. PeerJ. 2023;11:e15669. 37465158 10.7717/peerj.15669 PMC10351515

[4] Zulkiffli S, Yeoh OT, Yahya N, Kutty G. Evaluation and Comparison of Mechanical Properties of Lithium Disilicate-Based CAD/CAM Blocks. 2024; 53:217–29.

[5] Marchesi G, Camurri Piloni A, Nicolin V, Turco G, Di Lenarda R. Chairside CAD/CAM materials: current trends of clinical uses. Biology. 2021;10:1170. 34827163 10.3390/biology10111170 PMC8614873

[6] Sartori N, Tostado G, Phark J-H, Takanashi K, Lin R, Duarte S Jr. CAD/CAM High-Strength Glass-Ceramics. QDT. 2015; 38.

[7] Kenneth J, Anusavice D, Shen C, Rawls HR. Phillips’ Science of Dental Materials: Elsevier Health Sciences; 2012.

[8] Gracis S, Thompson V, Ferencz J, Silva N, Bonfante E. A New Classification System for All-Ceramic and Ceramic-like Restorative Materials. Int J Prosthodont. 2015;28:227–35. 25965634 10.11607/ijp.4244

[9] Anadioti E, Aquilino SA, Gratton DG, Holloway JA, Denry I, Thomas GW, Qian F. 3D and 2D marginal fit of pressed and CAD/CAM lithium disilicate crowns made from digital and conventional impressions. J Prosthodont. 2014;23:610–7. 24995593 10.1111/jopr.12180

[10] Sen N, Us YO. Mechanical and optical properties of monolithic CAD-CAM restorative materials. J Prosthet Dent. 2018;119:593–9. 28781072 10.1016/j.prosdent.2017.06.012

[11] Bindl A, Lüthy H, Mörmann W. Thin-wall ceramic CAD/CAM crown copings: strength and fracture pattern. J Oral Rehabil. 2006;33:520–8. 16774511 10.1111/j.1365-2842.2005.01588.x

[12] Brandt S, Winter A, Lauer H-C, Kollmar F, Portscher-Kim S-J, Romanos GE. IPS e. max for all-ceramic restorations: clinical survival and success rates of full-coverage crowns and fixed partial dentures. Materials. 2019;12:462. 30717358 10.3390/ma12030462 PMC6384731

[13] Fasbinder DJ, Dennison JB, Heys D, Neiva G. A clinical evaluation of chairside lithium disilicate CAD/CAM crowns. J Am Dent Assoc. 2010;141:S10–4. 10.14219/jada.archive.2010.0355 20516109

[14] Ku C-W, Park S-W, Yang H-S. Comparison of the fracture strengths of metal-ceramic crowns and three ceromer crowns. J Prosthet Dent. 2002;88:170–5. 12397244 10.1067/mpr.2002.127712

[15] French B, Jezek P, Appleman D. Virgilite; a new lithium aluminum silicate mineral from the Macusani glass, Peru. Am Min. 1978;63:461–5.

[16] Monmaturapoj N, Lawita P, Thepsuwan W. Characterisation and properties of lithium disilicate glass ceramics in the SiO 2-Li 2 OK 2 O-Al 2 O 3 system for dental applications. Adv Mater Sci Eng. 2013; 2013.

[17] Perez Canals M. Marginal Fit of CAD/CAM Crowns Milled From Two Different Ceramic Materials: Lithium Disilicate and Advanced Lithium Disilicate. University of Pittsburgh; 2023.

[18] Fasbinder DJ, CEREC Tessera TM Advanced Lithium Disilicate. 2021. https://assets.dentsplysirona.com/master/regions-countries/north-america/product-procedure-brand/cad-cam/CER-EN-US-document-White-Paper-CEREC-Tessera-1.pdf

[19] Hany C, Taymour M. Fracture resistance and failure mode of two restoration designs made of monolithic hybrid and glass machinable ceramics; in vitro study. Egypt Dent J. 2017;63:2771–83.

[20] Mullayousef HA. Mechanical and physical properties of three CAD/CAM glass-ceramics. 2022.

[21] Zhang Y, Vardhaman S, Rodrigues C, Lawn B. A critical review of dental lithia-based glass–ceramics. J Dent Res. 2023;102:245–53. 36645131 10.1177/00220345221142755 PMC9947811

[22] Lu Y, de Oliveira Dal Piva AM, Tribst JPM, Feilzer AJ, Kleverlaan CJ. Does glaze firing affect the strength of advanced lithium disilicate after simulated defects? Clin Oral Investig. 2023;27:6429–38. 37726488 10.1007/s00784-023-05246-1 PMC10630247

[23] Zhao T, Lian M-M, Qin Y, Zhu J-F, Kong X-G, Yang J-F. Improved performances of lithium disilicate glass-ceramics by seed induced crystallization. J Adv Ceram. 2021;10:614–26.

[24] Boitelle P, Tapie L, Mawussi B, Fromentin O. Evaluation of the marginal fit of CAD-CAM zirconia copings: Comparison of 2D and 3D measurement methods. J Prosthet Dent. 2018;119:75–81. 28461045 10.1016/j.prosdent.2017.01.026

[25] Azar B, Eckert S, Kunkela J, Ingr T, Mounajjed R. The marginal fit of lithium disilicate crowns: Press vs. CAD/CAM. Braz Oral Res. 2018;32:e001. 29364328 10.1590/1807-3107/2018.vol32.0001

[26] Faul F, Erdfelder E, Lang A-G, Buchner A. G* Power 3: A flexible statistical power analysis program for the social, behavioral, and biomedical sciences. Behav Res Methods. 2007;39:175–91. 17695343 10.3758/bf03193146

[27] Jalalian E, Zarbakhsh A, Khorshidi S, Golalipour S, Mohammadnasl S, Sayyari M. Comparative analysis of endocrown fracture resistance and marginal adaptation: CAD/CAM technology using lithium disilicate vs. zirconia-reinforced lithium silicate ceramics. Saudi Dent J. 2024;36:353–8. 38420004 10.1016/j.sdentj.2023.11.020 PMC10897595

[28] Petrie A, Sabin C. Medical statistics at a glance. Wiley; 2019.

[29] Phark JH, Duarte S Jr. Microstructural considerations for novel lithium disilicate glass ceramics: A review. J Esthet Restor Dent. 2022;34:92–103. 34995008 10.1111/jerd.12864

[30] Sheen C-Y, Dong J-K, Brantley WA, Han DS. A study of fracture loads and fracture characteristics of teeth. J Adv Prosthodont. 2019;11:187. 31297178 10.4047/jap.2019.11.3.187 PMC6609756

[31] Ferrini F, Paolone G, Di Domenico GL, Pagani N, Gherlone EF. SEM evaluation of the marginal accuracy of zirconia, lithium disilicate, and composite single crowns created by CAD/CAM method: comparative analysis of different materials. Materials. 2023;16:2413. 36984293 10.3390/ma16062413 PMC10058296

[32] Jakovac M, Klaser T, Radatović B, Skoko Ž, Pavić L, Žic M. Surface characterization and conductivity of two types of Lithium-based glass ceramics after accelerating ageing. Materials. 2020;13:5632. 33321786 10.3390/ma13245632 PMC7763873

[33] https://assets.dentsplysirona.com/master/regions-countries/north-america/product-procedure-brand/cad-cam/CER-EN-US-document-White-Paper-CEREC-Tessera-1.pdf [Accessed on: Nov, 2021] FDCTRWAf. In.

[34] Holst S, Karl M, Wichmann M, Matta R. A new triple-scan protocol for 3D fit assessment of dental restorations. Quintessence Int. 2011;42:651–7. 21842005

[35] Boitellea P, Tapieb L, Mawussic B, Fromentind O. 3D fitting accuracy evaluation of CAD/CAM copings–comparison with spacer design settings Evaluation 3D de l ‘exactitude de l ‘adaptation de chapes fabriquées par CFAO–Comparaison avec les valeurs de paramétrage du joint. Int J Comput Dent. 2016;19:27–43. 27027101

[36] Teixeira K, Duque T, Maia H, Gonçalves T. Fracture resistance and failure mode of custom-made post-and-cores of polyetheretherketone and nano-ceramic composite. Oper Dent. 2020;45:506–15. 32101501 10.2341/19-080-L

[37] Rizk A, El-Guindy J, Abdou A, Ashraf R, Kusumasari C, Eldin FE. Marginal adaptation and fracture resistance of virgilite-based occlusal veneers with varying thickness. BMC Oral Health. 2024;24:307. 38443910 10.1186/s12903-024-04071-6 PMC10913281

[38] Burke F. Maximising the fracture resistance of dentine-bonded all-ceramic crowns. J Dent. 1999;27:169–73. 10079622 10.1016/s0300-5712(98)00050-5

[39] Abo Elhassan RG, Nasr DM. Non-invasive digital technique for examination of marginal and internal adaptation of different ceramic table-tops. Egypt Dent J. 2024;70:1515–28.

[40] Farah RI, Alresheedi B. Evaluation of the marginal and internal fit of CAD/CAM crowns designed using three different dental CAD programs: A 3-dimensional digital analysis study. Clin Oral Investig. 2023;27:263–71. 36100722 10.1007/s00784-022-04720-6

[41] Zimmermann M, Ender A, Attin T, Mehl A. Fracture load of three-unit full-contour fixed dental prostheses fabricated with subtractive and additive CAD/CAM technology. Clin Oral Investig. 2020;24:1035–42. 31286262 10.1007/s00784-019-03000-0

[42] Zimmermann M, Egli G, Zaruba M, Mehl A. Influence of material thickness on fractural strength of CAD/CAM fabricated ceramic crowns. Dent Mater J. 2017;36:778–83. 28835598 10.4012/dmj.2016-296

[43] Aboudorra HA, Amr H, Hafez A, Hassan AA. Internal fit evaluation of all ceramic restoration fabricated by two CAD/CAM milling systems using cone beam CT (CBCT). Egypt Dent J. 2019;65:2467–79.

[44] Demirel M, Donmez MB. Fabrication trueness and internal fit of different lithium disilicate ceramics according to post-milling firing and material type. J Dent. 2024;144:104987. 38580056 10.1016/j.jdent.2024.104987

[45] Kojima K, Nagaoka K, Murata Y, Yamamoto K, Akiyama S, Hokii Y, Fusejima F. Marginal adaptation of CAD/CAM milled lithium disilicate glass ceramic crowns. J Osseointegration. 2022;14:201–4.

[46] Dolev E, Bitterman Y, Meirowitz A. Comparison of marginal fit between CAD-CAM and hot-press lithium disilicate crowns. J Prosthet Dent. 2019;121:124–8. 29961628 10.1016/j.prosdent.2018.03.035

[47] Salem NM, Kader SHA, Al Abbassy F, Azer AS. Evaluation of fit accuracy of computer-aided design/computer-aided manufacturing crowns fabricated by three different digital impression techniques using cone-beam computerized tomography. Eur J Prosthodont. 2016;4:32.

[48] Selvaraj H, Krithikadatta J, Shrivastava D, Onazi MAA, Algarni HA, Munaga S, Hamza MO, saad Al-fridy T, Teja KV, Janani K. Systematic review fracture resistance of endodontically treated posterior teeth restored with fiber reinforced composites-a systematic review. BMC Oral Health. 2023;23:566. 37574536 10.1186/s12903-023-03217-2 PMC10423428

[49] Peutzfeldt A, Sahafi A, Flury S. Bonding of restorative materials to dentin with various luting agents. Oper Dent. 2011;36:266–73. 21740244 10.2341/10-236-L

[50] Blatz M, Vonderheide M, Conejo J. The effect of resin bonding on long-term success of high-strength ceramics. J Dent Res. 2018;97:132–9. 28876966 10.1177/0022034517729134 PMC6429574

[51] Klosa K, Wolfart S, Lehmann F, Wenz H-J, Kern M. The effect of storage conditions, contamination modes and cleaning procedures on the resin bond strength to lithium disilicate ceramic. J Adhes Dent. 2009; 11. 19492714

[52] Balladares AO, Abad-Coronel C, Ramos JC, Fajardo JI, Paltán CA, Martín Biedma BJ. Comparative Study of the Influence of Heat Treatment on Fracture Resistance of Different Ceramic Materials Used for CAD/CAM Systems. Materials. 2024;17:1246. 38541399 10.3390/ma17061246 PMC10972524

[53] Rosentritt M, Schmid A, Huber C, Strasser T. In Vitro Mastication Simulation and Wear Test of Virgilite and Advanced Lithium Disilicate Ceramics. Int J Prosthodont. 2022;35:770–6. 35234750 10.11607/ijp.7820

[54] Jurado CA, Davila CE, Davila A, Hernandez AI, Odagiri Y, Afrashtehfar KI, Lee D. Influence of occlusal thickness on the fracture resistance of chairside milled lithium disilicate posterior full-coverage single‐unit prostheses containing virgilite: A comparative in vitro study. J Prosthodont. 2024. 10.1111/jopr.13870 PMC12541292 38790151

[55] Nouh I, Rafla N, Ebeid KK. Mechanical behavior of different machinable ceramic crowns using vertical and horizontal preparations: an in-vitro study. Braz Dent Sci. 2023; 26.

[56] Jurado CA, Bora PV, Azpiazu-Flores FX, Cho S-H, Afrashtehfar KI. Effect of resin cement selection on fracture resistance of chairside CAD-CAM lithium disilicate crowns containing virgilite: A comparative in vitro study. J Prosthet Dent. 2023. 10.1016/j.prosdent.2023.08.019 37739880

[57] Mohammad M. Fracture resistance of four different types of cad/cam lithium disilicate endocrowns. Egypt Dent J. 2023;69:1493–500.

[58] Hamza TA, Sherif RM. Fracture resistance of monolithic glass-ceramics versus bilayered zirconia‐based restorations. J Prosthodont. 2019;28:e259–64. 29044828 10.1111/jopr.12684

[59] Morsy N, Ghoneim MM, Ibrahim Y. Effect of cement spacer on fit accuracy and fracture strength of 3-unit and 4-unit zirconia frameworks. BMC Oral Health. 2024;24:586. 38773502 10.1186/s12903-024-04341-3 PMC11106921

