# Supplementary material for: Fit accuracy and fracture resistance evaluation of advanced lithium disilicate crowns (in- vitro study)
Source: BMC Oral Health. 2025 Jan 11;25:58. doi: 10.1186/s12903-024-05325-z (PMC11725217; doi:10.1186/s12903-024-05325-z)
Supplement: Supplementary file 1 — Supplementary Material 1 [file 12903_2024_5325_MOESM1_ESM.docx]

| **Material, instrument or equipment** | **Product name** | **Manufacturer and City** |
| --- | --- | --- |
| **Typodont ivory** | Nissin Typodont Teeth | Nissin dental products INC., Kyoto, Japan |
| **Acrylic Resin** | Acrostone | Acrostone Co., Cairo, Egypt |
| **Dublicating silicon materials** | Dupliflex | Dupliflex, Protechno, Spain |
| **Epoxy** | Kemapoxy 150 | Kemapoxy 150, CMB, Cairo, Egypt |
| **Dental restoration design software** | exocad Dental DB | exocad GmbH, Darmstadt, Germany |
| **dental restoration software** | MeditDesign v2.1 | MEDIT Corp, Seoul, Korea |
| **Medit lab scanner** | MEDIT T710 | MEDIT T710, MEDIT Corp, Seoul, Korea |
| **Ceramic furnace** | Programat P310 furnace | Ivoclar Vivadent AG, Schaan/Liechtenstein |
| **Universal testing machine** | Model 5ST | Model 5ST, Tinius Olsen, Redhill, Surrey, England. |
| **Stereo microscope** | Olympus | Olympus, B061, Tokyo, Japan. |
| **Scanning electron microscope** | JSM-IT200 | Jeol JSM-IT200; Jeol Ltd. Akishima, Tokyo, Japan |
